# Enhancing Virus-Specific Immunity *In Vivo* by Combining Therapeutic Vaccination and PD-L1 Blockade in Chronic Hepadnaviral Infection

**DOI:** 10.1371/journal.ppat.1003856

**Published:** 2014-01-02

**Authors:** Jia Liu, Ejuan Zhang, Zhiyong Ma, Weimin Wu, Anna Kosinska, Xiaoyong Zhang, Inga Möller, Pia Seiz, Dieter Glebe, Baoju Wang, Dongliang Yang, Mengji Lu, Michael Roggendorf

**Affiliations:** 1 Institute for Virology, University Hospital of Essen, University of Duisburg-Essen, Essen, Germany; 2 Hepatology Unit and Department of Infectious Diseases, Nanfang Hospital, Southern Medical University, Guangzhou, China; 3 Institute for Medical Virology, Justus-Liebig University Giessen, Giessen, Germany; 4 Department of Infectious Diseases, Union Hospital, Tongji Medical College, Huazhong University of Science and Technology, Wuhan, China; The Rockefeller University, United States of America

## Abstract

Hepatitis B virus (HBV) persistence is facilitated by exhaustion of CD8 T cells that express the inhibitory receptor programmed cell death-1 (PD-1). Improvement of the HBV-specific T cell function has been obtained *in vitro* by inhibiting the PD-1/PD-ligand 1 (PD-L1) interaction. In this study, we examined whether *in vivo* blockade of the PD-1 pathway enhances virus-specific T cell immunity and leads to the resolution of chronic hepadnaviral infection in the woodchuck model. The woodchuck PD-1 was first cloned, characterized, and its expression patterns on T cells from woodchucks with acute or chronic woodchuck hepatitis virus (WHV) infection were investigated. Woodchucks chronically infected with WHV received a combination therapy with nucleoside analogue entecavir (ETV), therapeutic DNA vaccination and woodchuck PD-L1 antibody treatment. The gain of T cell function and the suppression of WHV replication by this therapy were evaluated. We could show that PD-1 expression on CD8 T cells was correlated with WHV viral loads during WHV infection. ETV treatment significantly decreased PD-1 expression on CD8 T cells in chronic carriers. *In vivo* blockade of PD-1/PD-L1 pathway on CD8 T cells, in combination with ETV treatment and DNA vaccination, potently enhanced the function of virus-specific T cells. Moreover, the combination therapy potently suppressed WHV replication, leading to sustained immunological control of viral infection, anti-WHs antibody development and complete viral clearance in some woodchucks. Our results provide a new approach to improve T cell function in chronic hepatitis B infection, which may be used to design new immunotherapeutic strategies in patients.

## Author Summary

Chronic hepatitis B virus (HBV) infection is still one of the major public health problems. Two billion people worldwide have been infected with HBV, of whom more than 360 million developed chronic infection. Every year, approximately one million of these individuals will die from HBV-associated liver diseases such as cirrhosis and hepatocellular carcinoma (HCC). Treatment of chronic hepatitis B remains a clinical challenge, and alternative strategies to treat chronic HBV infection are urgently needed. Here, we designed a new combination strategy to enhance the patient's own antiviral immune response and to achieve long-term viral suppression. The therapeutic effect of our combination therapy strategy for chronic hepadnaviral infection was tested in the woodchuck model. We demonstrated that our novel combination therapy could elicit potent antiviral immune response and achieved a strong antiviral effect, leading to sustained immunological control of chronic hepadnaviral infection and complete viral clearance in treated woodchucks. The results of this study may have an impact on clinical trials of the immunotherapy in chronically HBV-infected patients.

## Introduction

Hepatitis B virus (HBV) infection evolves into a chronic liver disease and leads to severe sequelae in about 5% of infected adults and in a larger proportion of children. It is estimated that approximately 400 million people are chronically infected with HBV worldwide. There are two types of antiviral therapies currently available for chronic HBV: treatment with pegylated interferon alpha (PEG-IFNα) and nucleot(s)ide analogues, such as entecavir (ETV) and tenofovir. However, treatment with PEG-IFNα leads to a sustained antiviral response in only about 30% patients and is associated with side effects. The introduction of PEG-IFNα in combination with nucleoside analogues did not significantly increase the rate of sustained responders [1], [2]. Although treatment with nucleoside analogues improves the clinical condition of chronic HBV patients, it is hampered by emergence of drug resistance mutations, and rebounding viremia after cessation of antiviral therapy [3], [4]. Therefore, alternative strategies to treat chronic HBV infection are urgently needed.

Persistent HBV infection is associated with functional exhaustion of virus-specific CD8 T cells [5]. This defect in virus-specific T cells is one of the primary reasons for the inability of the host to eliminate the persisting pathogen. Therefore, therapeutic vaccination, which aims to enhance the patient's own antiviral cellular immune response, has been considered as an alternative therapy. However, the efficacy of such strategies in patients has so far been disappointing [6], [7], [8]. Recent work suggests that the high viral load at the time of vaccination might explain the inefficient responses to therapeutic vaccination [9], [10]. Thus, it is important to develop a therapeutic vaccine strategy which could effectively boost endogenous T cell responses to control persistent viral infections.

Recent studies in chronic virus infection models indicate that the interaction between the inhibitory receptor programmed death-1 (PD-1) on lymphocytes and its ligands plays a critical role in T-cell exhaustion [11], [12], [13], [14]. In various human chronic infections, including HBV, high PD-1 levels are expressed by virus-specific T cells, and improvement of the T-cell function has been obtained *in vitro* by inhibition of the PD-1/PD-ligand 1 (PD-L1) interaction [15], [16], [17], [18], [19], [20], [21]. Moreover, *in vivo* blockade of PD-1/PD-L1 pathway has successfully been applied in mice persistently infected with lymphocytic choriomeningitis virus (LCMV) to restore the antiviral function of exhausted T cells, and hence improved the effect of the therapeutic vaccination [11], [22].

We have previously demonstrated that therapeutic DNA vaccines in combination with an antiviral nucleoside analogue result in a prolonged suppression of WHV replication in chronically WHV infected woodchucks [23]. Recently, we have also demonstrated that *in vitro* blockade of the woodchuck PD-1/PD-L pathway could restore the T cell functions in chronic WHV infection [24]. In this study, we examined whether *in vivo* blockade of the PD-1 pathway in combination with antiviral nucleoside analogue treatment and therapeutic vaccination could enhance CD8 T cell immunity and lead to the resolution of chronic WHV infection in the woodchuck model. Persistently WHV-infected woodchucks were first treated with antiviral drug ETV to decrease the viral replication, and then received therapeutic vaccination and PD-L1 antibody treatment. This combinatorial therapeutic vaccination potently enhanced WHV-specific CD8 T cell responses, resulted in absence of WHV DNA in plasma and seroconversion to anti-WHs in two animals. However, residual WHV replication was still detectable in the liver of some animals.

## Results

### Molecular cloning and characterization of woodchuck PD-1

**Figure 1:**
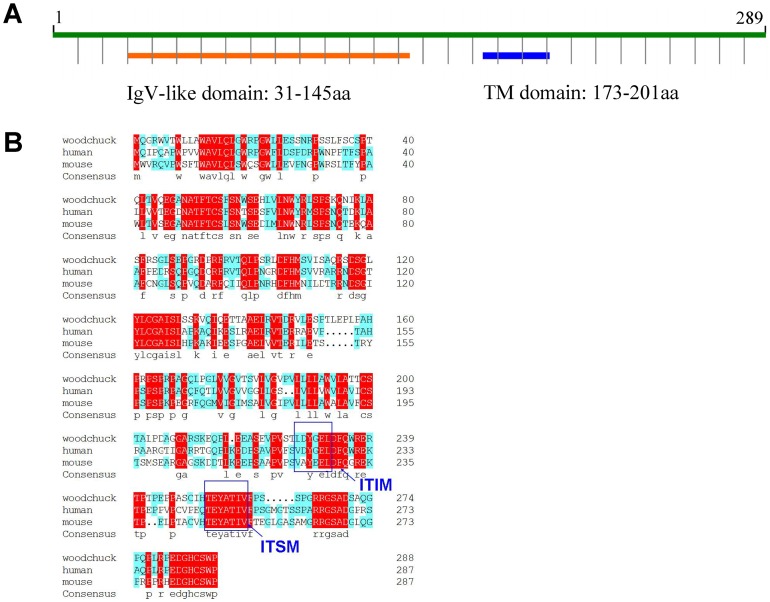
Analysis of the deduced amino acid sequence of woodchuck PD-1. (A) Predicted secondary structure of woodchuck PD-1. The locations of immunoglobulin V-like domain (Ig-V-like domain) and transmembrane region (TM domain) are indicated. (B) Alignment of woodchuck PD-1 with human PD-1 and mouse PD-1. Consensus sequences are marked with red. Blue boxes indicate the conserved ITIM and ITSM motifs.

The complete coding region of woodchuck PD-1 was obtained by reverse transcription polymerase chain reaction (RT-PCR) and subjected to sequence analysis. A comparison of the woodchuck PD-1 sequence revealed a high homology at the nucleotide (nt) and amino acid (aa) levels to the counterparts of mammalian species (Table 1). The length of putative woodchuck PD-1 protein is 288 aa residues, which shows the typical features of a membrane protein, an extracellular domain with a Ig-V-like region, a trans-membrane domain and a cytoplasmic domain (Figure 1A). Importantly, the cytoplasmic domain of woodchuck PD-1 contains two highly conserved structural motifs, an immunoreceptor tyrosine-based inhibition motif (ITIM) and an immunoreceptor tyrosine-based switch motif (ITSM) (Fig. 1B). The ITSM of PD-1 is believed to be essential for the inhibitory function of mouse PD-1 and human PD-1 [25], [26]. Therefore, this result indicates that woodchuck PD-1 also behaves as an inhibitory molecule in the woodchuck immune system.

**Table 1:** Homology comparison of woodchuck, human and mouse PD-1.

Species	Woodchuck	Human	Mouse
Woodchuck	100 *(100)*	–	–
Human	74.71 *(62.98)*	100 *(100)*	–
Mouse	71.95 *(58.82)*	70.93 *(59.72)*	100 *(100)*

The numbers outside of brackets indicate the similarities at nucleotide level in percentage; the numbers inside of brackets indicate the similarities at the amino acid level in percentage.

### PD-1 expression on CD8 T cells during acute WHV infection

**Figure 2:**
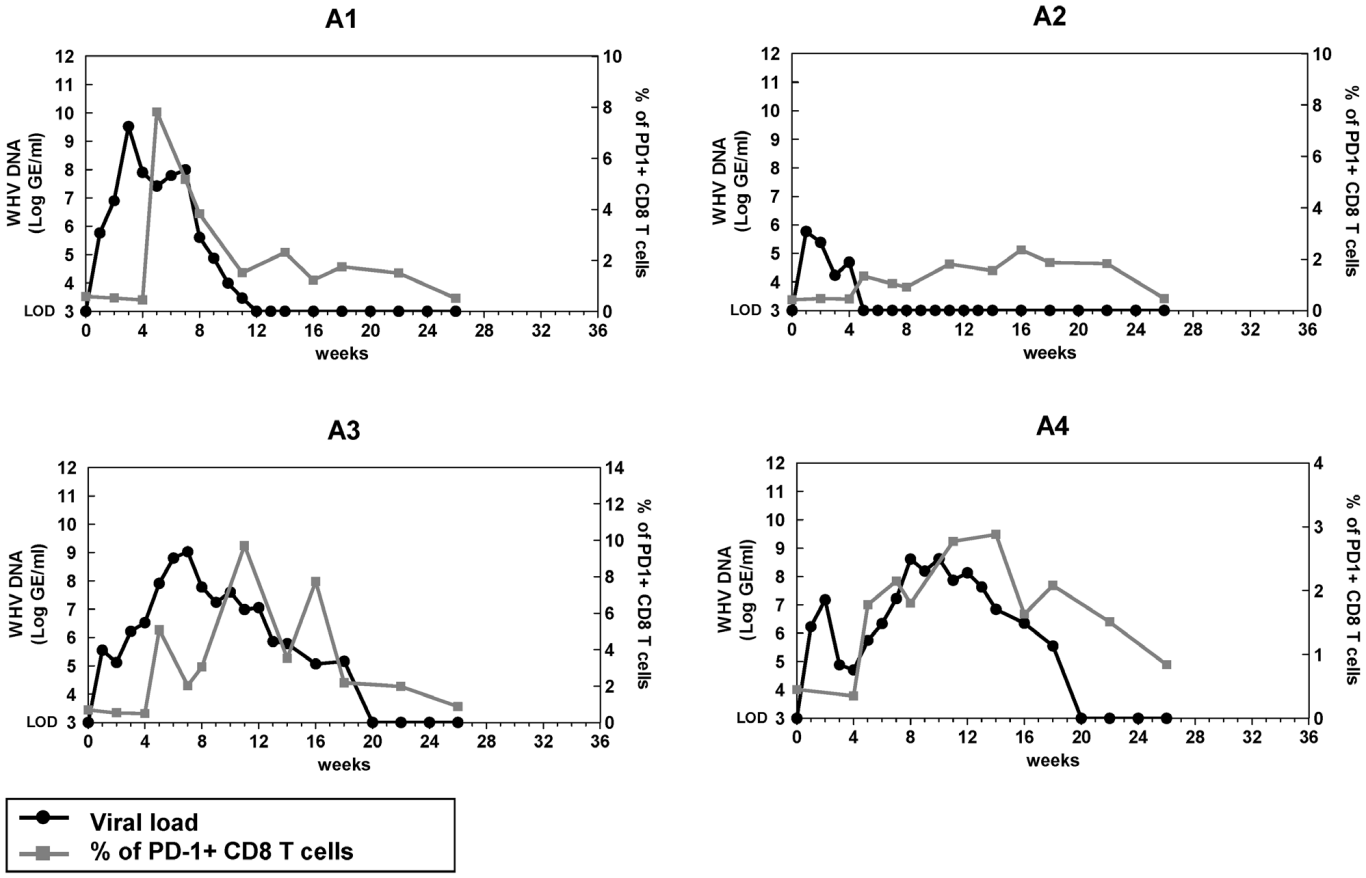
Kinetics of PD-1 expression on CD8 T cells during acute WHV infection. Four adult woodchucks were inoculated with 1×10^7^ or 1×10^9^ WHV genome equivalents. Sera and PBMCs of infected woodchucks were freshly isolated at indicated time points. WHV viral loads (black dots) were quantified by real-time PCR, and percentages of PD-1+ CD8 (CD3+ CD4−) T cells (gray squares) were analyzed by FACS. LOD: lower limit of detection.

It has been reported that PD-1 expression is up-regulated on HBV-specific CD8 T cells in the early phase of acute HBV infection [27]. Therefore, we first determined the kinetics of PD-1 expression on CD8 T cells (CD3+ CD4−) throughout the course of acute WHV infection in the woodchuck model. Four adult woodchucks were inoculated with 1×10^7^ or 1×10^9^ WHV genome equivalents and all of them went through an acute resolving infection. PD-1 expression on CD8 T cells was significantly up-regulated in acute WHV infection and reached its peak when the viremia started to decline. Following the successful viral clearance, PD-1 expression continuously decreased to the levels detected prior to WHV infection (Figure 2). One woodchuck (A2) with very short and low viremia showed almost no increase of PD-1 expression on CD8 T cells (Figure 2). These data clearly indicate that the expression of PD-1 on CD8 T cells is associated with WHV viremia in acute resolving WHV infection.

### PD-1 expression on peripheral blood mononuclear cells (PBMCs) and T cells during chronic WHV infection

**Figure 3:**
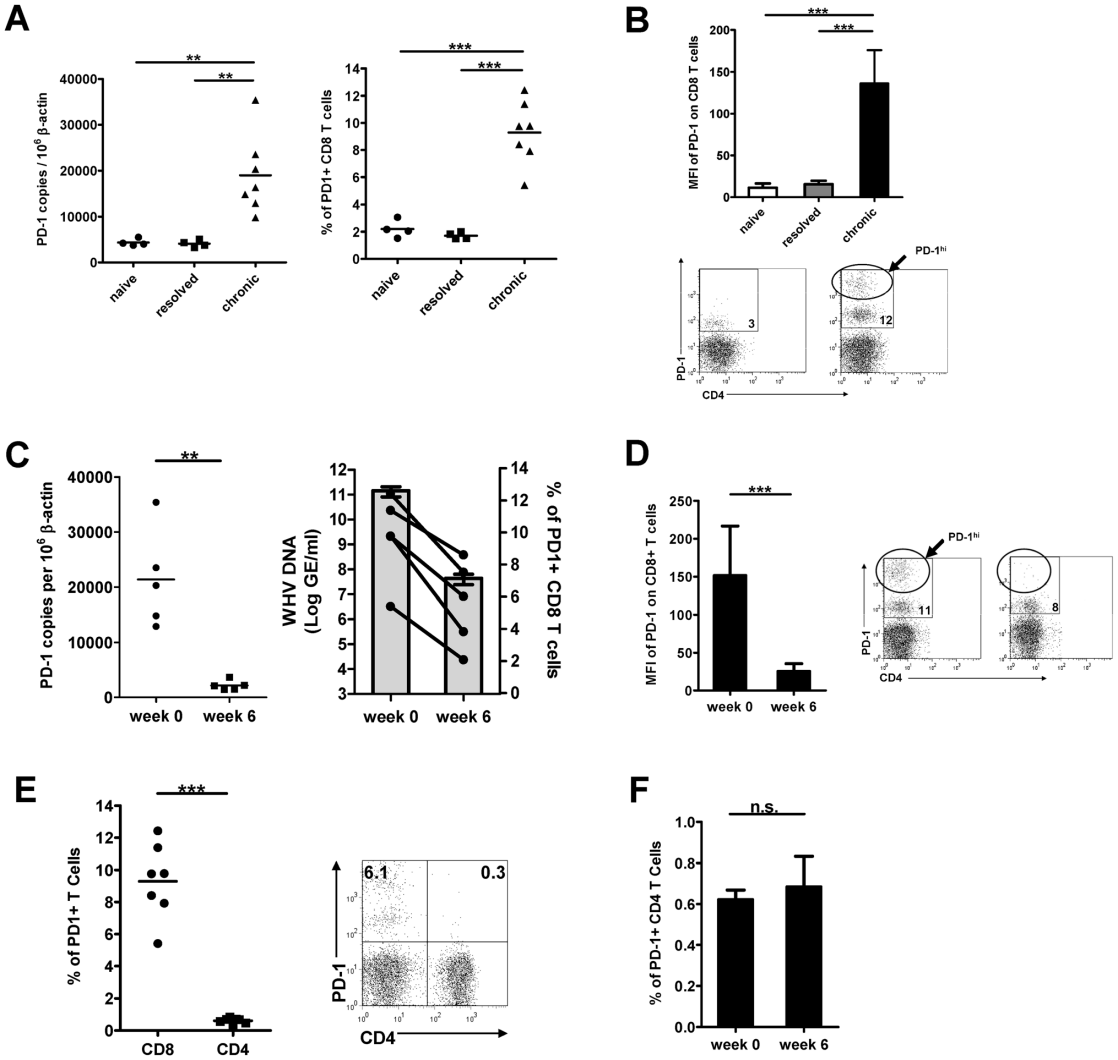
Expression of PD-1 on PBMCs and T cells during chronic WHV infection. (A) PBMCs were isolated from naïve (n = 4), WHV infection resolved (n = 4), and untreated chronically WHV infected woodchucks (n = 7). The absolute copy numbers of PD-1 mRNA of total PBMCs were analyzed by real-time PCR. The percentages of PD-1+ CD8 (CD3+ CD4−) T cells were analyzed by FACS. (B) The mean fluorescence intensity (MFI) of PD-1 expression on CD8 T cells was calculated and compared between different groups. Representative dot plot shows the population of PD-1^hi^ CD8 T cells of chronically WHV infected woodchucks. (C) PD-1 expression in chronically WHV infected woodchucks before and 6 weeks after ETV treatment (n = 5) were compared. Left: The absolute copy numbers of PD-1 mRNA of total PBMCs were analyzed by real-time PCR. Right: The percentages of PD-1+ CD8 T cells (black dots) are coordinated with the WHV viral loads (gray columns) in ETV treated woodchucks. (D) The changes of mean fluorescence intensity (MFI) of PD-1 expression on CD8 T cells before and 6 weeks after ETV treatment were compared. Representative dot plot shows the disappearance of PD-1^hi^ CD8 T cells in chronically WHV infected woodchucks after ETV treatment. (E) The percentages of PD-1+ CD4 T cells (CD3+ CD4+) were analyzed by FACS and compared to that of CD8 T cells (CD3+ CD4−). Representative dot plot shows the populations of PD-1+ CD4 T cells and PD-1+ CD8 T cells of chronically WHV infected woodchucks. (F) The percentages of PD-1+ CD4 T cells of chronically WHV infected woodchucks before and after ETV treatment (n = 5) were compared.

Next, we examined the level of PD-1 expression on PBMCs and T cells in woodchucks with chronic WHV infection. The PD-1 mRNA level of PBMCs and the percentage of PD-1+ CD8 T cells in chronic carriers were significantly higher than that of naïve woodchucks or resolvers (Figure 3A). The mean fluorescence intensity (MFI) of PD-1 expression on CD8 T cells in chronic carriers was also significantly increased. Indeed, a distinct population of CD8 T cells which expressed extremely high levels of PD-1 (PD-1^hi^) was observed in the PBMCs of those woodchucks (Figure 3B). We also compared PD-1 expression on PBMCs and CD8 T cells prior to antiviral treatment and at week 6 of antiviral treatment. Before initiation of ETV therapy, high levels of PD-1 expression were detected on both PBMCs and CD8 T cells in all subjects analysed. Antiviral treatment resulted in dramatic decline of detectable serum viral load, coincident with decrease of the PD-1 expression on PBMCs and CD8 T cells at both mRNA and protein levels (Figure 3C). The MFI of PD-1 on CD8 T cells also decreased dramatically, and the PD-1^hi^ CD8 T cells almost vanished after ETV treatment (Figure 3D). These data indicate that high levels of antigen in chronic WHV infection may drive continuous high-level expression of PD-1 on CD8 T cells. The suppression of WHV replication resulted in a relatively lower level of PD-1 expression, which may facilitate the restoration of T cell function.

The PD-1 expression on CD4 T cells was quite low in contrast to that on CD8 T cells in woodchucks with chronic WHV infection. Only a small number of CD4 T cells were found PD-1 positive (Figure 3E). Antiviral ETV treatment showed no influence on PD-1 expression on CD4 T cells (Figure 3F).

### *In vivo* PD-L1 blockade enhances WHV-specific T cell immunity induced by therapeutic vaccination during chronic WHV infection

**Figure 4:**
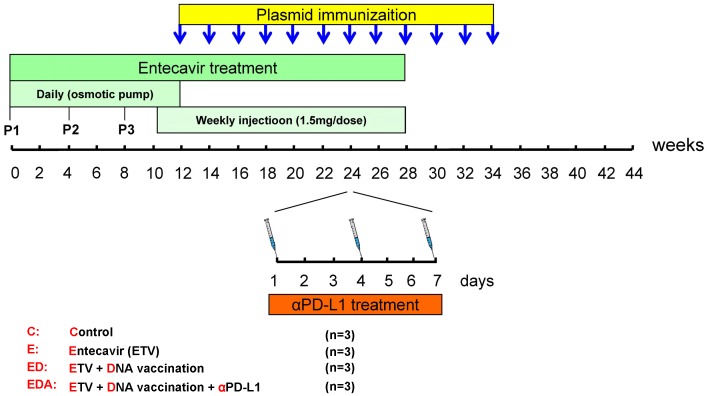
Schema of triple combination therapy of ETV treatment, DNA vaccination and *in vivo* PD-L1 blockade. The antiviral drug ETV was administered for 28 weeks to suppress the WHV replication. Initially, the drug was administered for 12 weeks by using osmotic pumps which were implanted surgically under the skin of the animals. Pumps were exchanged every 4 weeks and overall 3 pumps were implanted for each woodchuck. P1, P2 and P3 mean the timings of the pump implantations. From week 10 to 28, subcutaneous injections of 1.5-1/PD-L1 pathway blockade, woodchucks were treated with rabbit polyclonal PD-L1 blocking antibody (αPD-L1) 3 times in week 24. Four groups of woodchucks (n = 3/group) were included. **C**: control group without any treatment; **E**: ETV treated only group; **ED**: ETV in combination with DNA vaccinations group; **EDA**: ETV and DNA vaccination in combination with anti-PDL1 antibody treatment group.

To evaluate the effect of *in vivo* PD-L1 blockade during chronic WHV infection, a triple combination therapy strategy combined of antiviral treatment, therapeutic vaccination and PD-L1 antibody blocking was designed (Figure 4). The antiviral drug ETV was administered for 28 weeks to suppress the WHV replication. Starting from week 12, animals received subsequently 12 intramuscular immunizations with DNA plasmids, expressing WHV core antigen (WHcAg) and surface antigen (WHsAg). For PD-1/PD-L1 pathway blockade, woodchucks were treated with rabbit polyclonal PD-L1 blocking antibody (αPD-L1) 3 times in week 24. In total 12 chronically WHV-infected woodchucks were included in this experiment and were divided into four differently treated groups (Figure 4).

**Figure 5:**
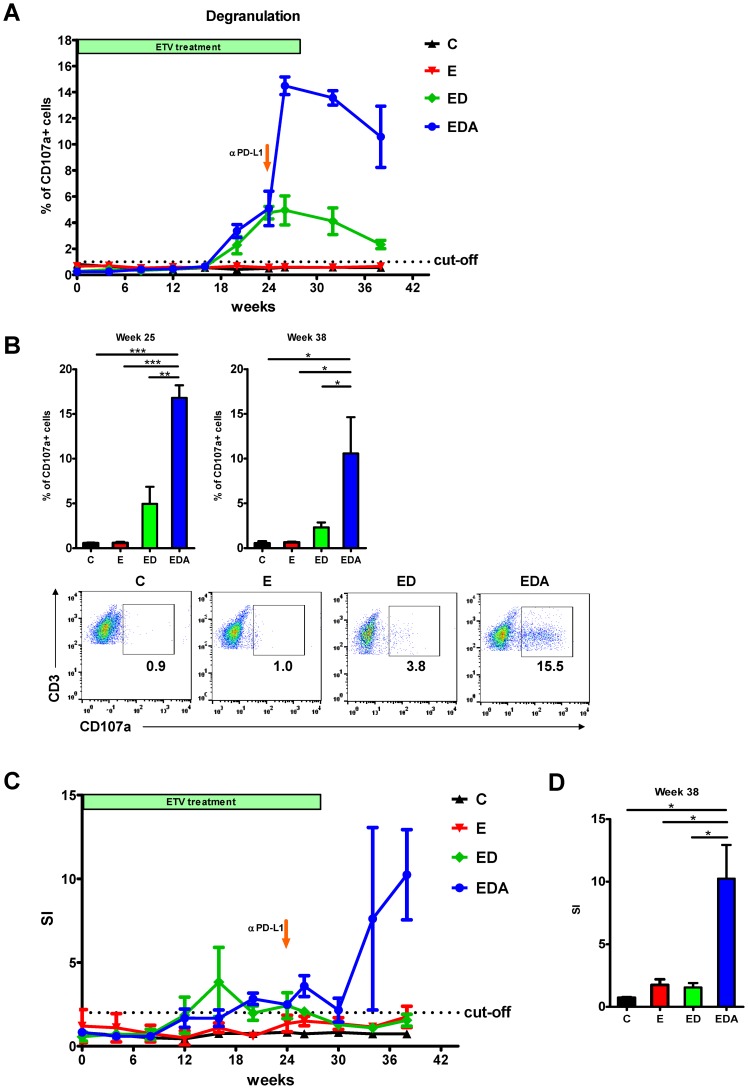
*In vivo* PD-L1 blockade synergizes with therapeutic vaccination to enhance WHV-specific T cell immunity. (A) WHcAg-specific T cell responses of differently treated woodchucks were analyzed by CD107a degranulation assay. The kinetics of WHcAg-specific CD8 T cell response of 4 differently treated groups of woodchucks is presented. **C**: control group without any treatment; **E**: ETV treated only group; **ED**: ETV in combination with DNA vaccinations; **EDA**: ETV and DNA vaccination in combination with anti-PDL1 antibody treatment. (B) Upper: Comparison of the strength of WHcAg-specific T cell response of woodchucks with different treatments at week 25 and week 38. Lower: Representative dot plots show the population of CD107a+ CD8 T cells of treated woodchuck PBMCs in response to WHcAg-specific peptide stimulation (week 25). (C) WHcAg-specific T cell responses of differently treated woodchucks were analyzed by proliferation assay. The kinetics of PBMCs proliferation in response to WHV core protein stimulation of woodchucks with different treatments is presented. (D) Comparison of the strength of PBMCs proliferation of woodchucks with different treatments at week 38. Stimulation index (SI) is calculated with the formula: (stimulated cpm - blank cpm)/(unstimulated cpm - blank cpm).

Firstly, we longitudinally monitored individual woodchucks for WHcAg-specific and WHsAg-specific T cell responses by flow cytometric CD107a assay. Consistent with our previous study [28], [29], the WHcAg-specific and WHsAg-specific degranulation were not detectable in the chronic carriers without treatment or with only ETV treatment. Therapeutic vaccination in combination with antiviral treatment could induce a slight expansion of the WHcAg-specific CD8 T cell population in those animals after week 20. Importantly, *in vivo* PD-L1 blockade in the vaccinated woodchucks resulted in a significant increase in the WHcAg-specific CD8 T cell response (Figure 5A). The enhancement of WHcAg-specific CD8 T cell response in woodchucks of triple combination therapy group was observed immediately after αPD-L1 administration (one week). Moreover, this effect was sustained even after cessation of the αPD-L1 treatment. At week 38, which was 14 weeks after the cessation of the αPD-L1 treatment, strong WHcAg-specific T cell responses could still be detected in αPD-L1 treated woodchucks (Figure 5B). Compared to the WHcAg-specific T cell response, the WHsAg-specific CD8 T cell response induced by DNA vaccination in ETV treated woodchucks was weak. Additional PD-L1 blockade did not result in any significant increase in the WHsAg-specific CD8 T cell response (Figure S1).

We also longitudinally monitored individual woodchucks for WHcAg-specific responses by 2[^3^H]-adenine-based proliferation assay. In response to WHV core protein stimulation, no proliferation of PBMCs was observed in the chronic carriers without treatment or with only ETV treatment, and only weak proliferation of PBMCs was observed in woodchucks received DNA vaccination in combination with ETV treatment. In contrast to that, woodchucks received additional PD-L1 blockade showed strong proliferation of their PBMCs in response to WHV core protein stimulation after the αPD-L1 administration (Figure 5C and 5D, Table S1).

### *In vivo* PD-L1 blockade in combination with therapeutic vaccination achieves enhanced viral control during chronic WHV infection

To examine whether our triple combination therapy resulted in better control of viral infection, we monitored the WHV viremia, WHsAg levels in serum, development of anti-WHs, viral replication in the liver and liver transaminase levels of the treated woodchucks.

**Figure 6:**
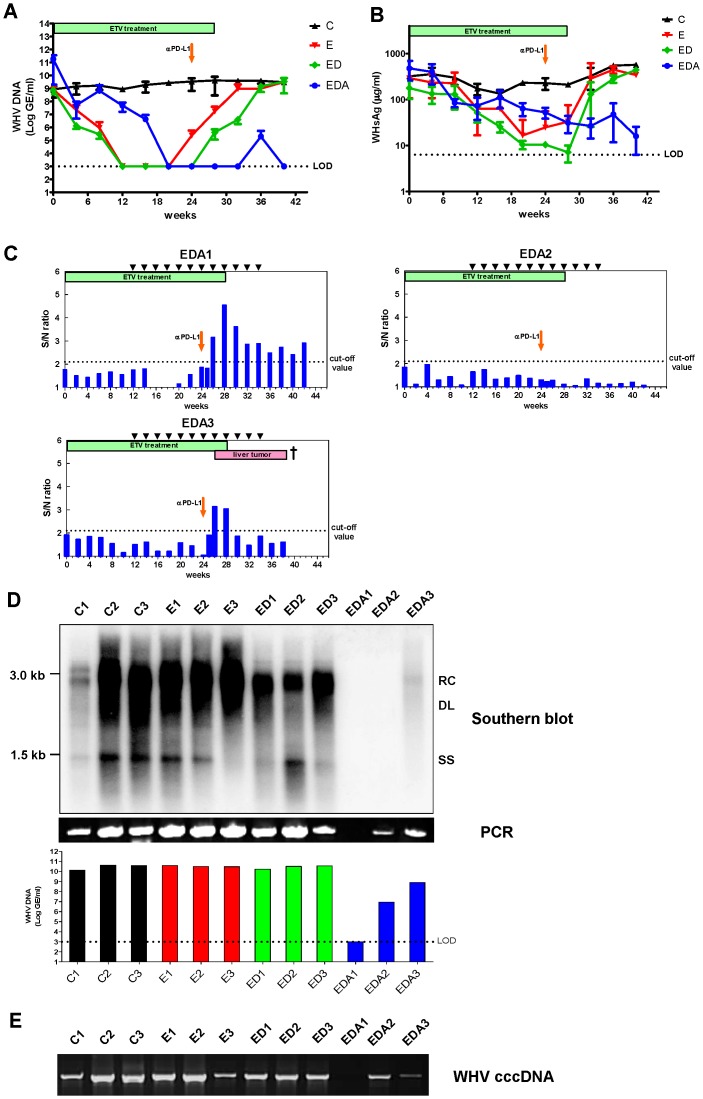
*In vivo* PD-L1 blockade synergizes with therapeutic vaccination to control WHV replication. Serum WHV DNA (A) and WHsAg (B) concentrations of different treatment groups are presented at indicated time points. **C**: control group without any treatment; **E**: ETV treated only group; **ED**: ETV in combination with DNA vaccinations; **EDA**: ETV and DNA vaccination in combination with anti-PDL1 antibody treatment. LOD: lower limit of detection. (C) The kinetics of serum anti-WHs antibodies levels of woodchucks with triple combination treatment is presented. Anti-WHs antibodies level was determined using the following formula: S/N ratio = sample OD value/negative control OD value. Cut-off value of S/N ratio was 2.1. (D) Detection of WHV replication intermediates in liver tissues of differently treated woodchucks. Liver samples were taken 14 weeks after cessation of ETV treatment. Total DNA were extracted from the liver samples and subjected to Southern blotting (upper), PCR (middle), and realtime PCR (lower). RC: relaxed circular DNA, DL: double stranded linear DNA, SS: single stranded DNA. (E) Detection of WHV cccDNA in liver tissues of differently treated woodchucks. Extracted liver DNA samples were digested with Plasmid-Safe ATP-Dependent DNase and subjected cccDNA-specific PCR with primers amplifying the WHV gap-spanning region.

The basal WHV DNA loads in the serum prior to ETV therapy in WHV chronic carriers enrolled in the experiment ranged from 4.27×10^6^ GE/ml to 3.73×10^11^ GE/ml. Consistent with our previous study [30], the treatment with 0.5 mg ETV/kg body weight led to a significant reduction of the serum WHV DNA concentrations up to >6 log (Figure 6A). In woodchucks that received only ETV, the viral rebound was observed after week 24, and WHV DNA concentrations returned to the pre-treatment levels shortly after cessation of ETV treatment. In woodchucks that received ETV treatment in combination with therapeutic vaccination, the relapse of viremia was delayed and the serum WHV DNA levels remained undetectable in all animals till week 28. However, the viral rebound occurred in these woodchucks after cessation of ETV treatment, and WHV DNA concentrations returned to the pre-treatment levels at the end of the observation period. In contrast to that, the serum WHV DNA levels of woodchucks which received additional αPD-L1 treatment remained undetectable at almost all time points till the end of the observation period (Figure 6A). There was a relapse of viremia in only one αPD-L1 treated woodchuck (EDA3) after week 36. This woodchuck had developed massive liver tumors during the ETV treatment (Figure S2) and had to be sacrificed before the end of the observation period.

The initial levels of WHsAg in the sera varied between the individual animals and were ranging from 45.8 µg/ml to 698.3 µg/ml. A significant decrease of WHsAg was observed in all woodchucks during the ETV treatment. In the woodchucks treated with only ETV or ETV in combination with DNA vaccination, WHsAg concentrations returned to the pre-treatment levels shortly after cessation of ETV treatment. In contrast to that, woodchucks that received additional αPD-L1 treatment showed no relapse of WHsAg in the sera after cessation of ETV treatment (Figure 6B). Notably, woodchuck EDA1 which was negative for WHV DNA in both serum and liver was also WHsAg negative (below limit of detection after week 28). However, WHsAg of the other 2 woodchucks (EDA2 and EDA3) with αPD-L1 treatment remained at very low levels till the end of our observation (Figure S3). No anti-WHs antibody was detectable in woodchucks of the ETV treatment only group and of the DNA vaccination group (data not shown). In contrast, 2 woodchucks of the PD-L1 blockade group developed anti-WHs antibodies. These 2 woodchucks became anti-WHs antibody positive 2 weeks after the PD-L1 blockade. The titer of anti-WHs antibodies in woodchuck EDA1 was high, and remained positive till the end of the observation. The titer in the other woodchuck EDA3 was relatively lower, and dropped very quickly accomplished with a massive hepatocellular carcinoma (HCC) development. Woodchuck EDA2 did not develop a detectable level of anti-WHs antibody within the observation period up to week 42 (Figure 6C). Anti-WHc antibodies in the sera of treated woodchucks were also examined by semi-quantitative ELISA. All chronic carriers showed fluctuant anti-WHc levels in the sera throughout the whole observation period. No significant change of serum anti-WHc levels was observed after PD-L1 antibodies administration in treated woodchucks (Figure S4).

In addition to serum levels of WHV DNA, the WHV replication in the livers of treated woodchucks was evaluated by Southern blot. Woodchucks of the untreated group C, the ETV treated only group E and the ETV plus DNA vaccination group ED showed obvious WHV replication in the liver at the end of the observation period up to week 42. In contrast, woodchuck EDA1 and EDA2 of the triple combination therapy group did not show any WHV replication in the liver at week 42. Woodchuck EDA3 also demonstrated a very low level of WHV replication (Figure 6D). In addition, we analyzed the presence of WHV DNA in the liver by sensitive PCR. Consistent with the results of Southern blot, woodchuck EDA1 was negative for WHV DNA in the liver. Woodchuck EDA2 and EDA3 also showed reduced WHV DNA loads in the liver compared to those of woodchucks without αPD-L1 treatment (Figure 6D). Realtime PCR was also performed to precisely quantify the amounts of WHV DNA in the liver of all animals. The lower detection limit of this realtime PCR is 1000 GE/ml. The liver WHV DNA loads of animals without αPD-L1 treatment ranged from 1.34×10^10^ GE/ml to 4.33×10^10^ GE/ml. In contrast, in animals received additional αPD-L1 treatment, two woodchucks (EDA2 and EDA3) showed 2–4 log reduction of the liver WHV DNA concentrations, and one woodchuck (EDA1) remained undetectable for liver WHV DNA (Figure 6D).

Covalently closed circular DNA (cccDNA) is responsible for persistence of infection in the natural course of chronic HBV infection and during prolonged antiviral therapy. Therefore, the effect of the different treatment regimens on the cccDNA pool in the liver was examined. Consistent with the results of WHV DNA detection in the liver, woodchucks of the untreated group, the ETV treated only group and the ETV plus DNA vaccination group showed obvious WHV cccDNA presence in the liver at the end of the observation period up to week 42. In contrast, WHV cccDNA remained undetectable in the liver of woodchuck EDA1. Woodchuck EDA2 and EDA3 also showed reduced cccDNA loads in the liver compared to those of woodchucks without αPD-L1 treatment (Figure 6E).

**Figure 7:**
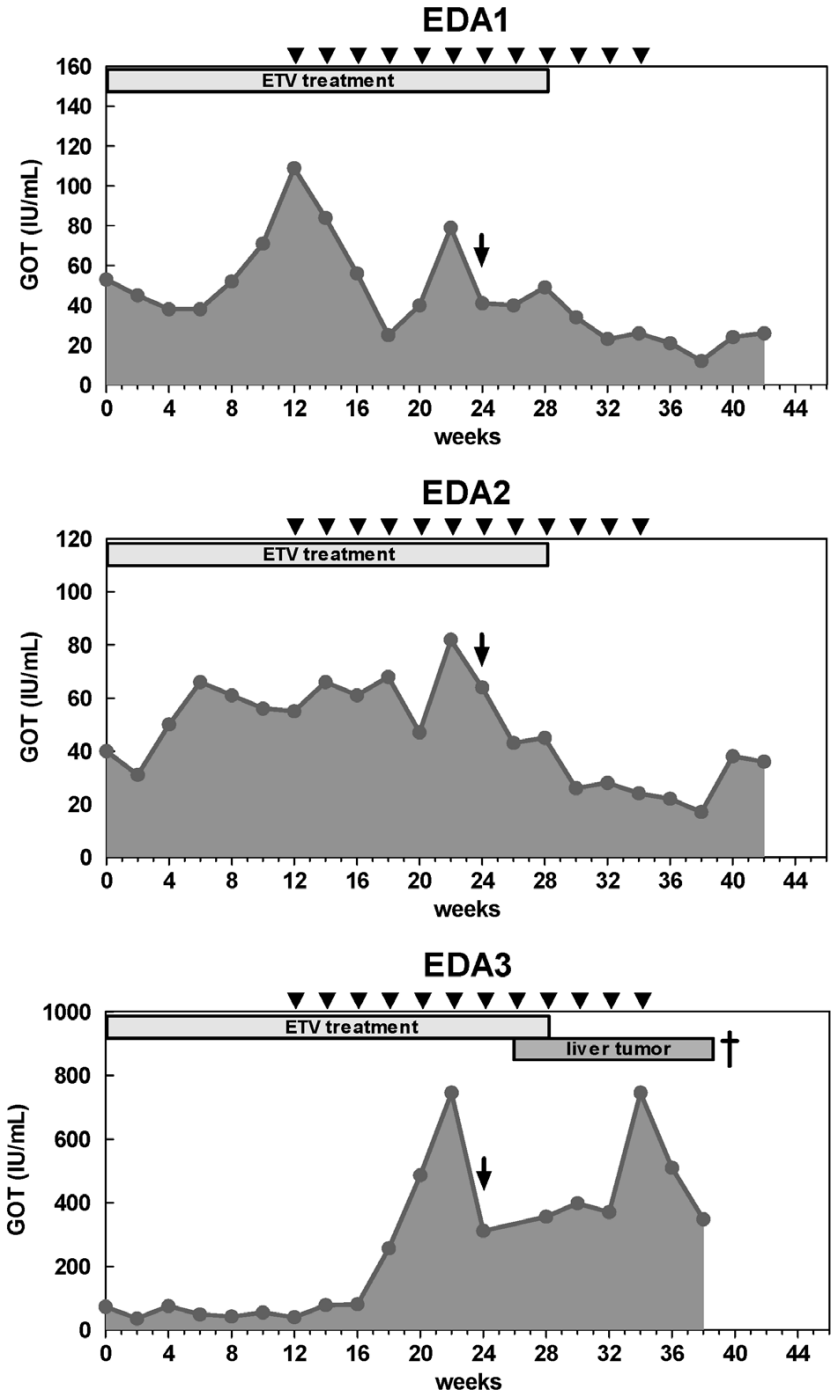
Liver transaminase GOT levels of woodchucks with combination treatment. The GOT values (gray areas) of the woodchuck sera were measured up to the end of the observation period. The duration of ETV treatment is indicated with a gray box, DNA vaccinations are indicated with black triangles, and αPD-L1 treatment is indicated with a black arrow. WHcAg-specific CD8 T cell responses of the woodchucks are indicated with black bars.

Liver transaminase GOT levels were measured during ETV treatment, immunizations and antibody treatment. In most of the treated woodchucks, GOT flares were apparently associated with a reduction of WHV DNA loads. This is likely due to a partial restoration of immunological functions, similar to the reported cases in clinical trials of ETV. Although PD-L1 blockade strongly restored the T cell function in antibody treated woodchucks, GOT levels of these animals after antibody injection did not increase (Figure 7). GOT flare was observed after PD-L1 blockade in only one woodchuck (EDA3), but this elevation is more likely due to the development of massive HCC in this animal.

### The triple combination regimen breaks the immune tolerance against WHV antigens and leads to viral load reduction in WHV transgenic mice

We also evaluated the effect of our triple combination regimen on the induction of WHV-specific CD8 T cell response and the control of WHV replication in 1217 WHV transgenic (tg) mice carrying a 1.3 fold overlength WHV transgenome [30]. The antiviral drug ETV was daily administered for 10 days to suppress the WHV replication in mice. For DNA vaccination, mice received two intramuscular immunizations with DNA plasmid expressing WHcAg on day 7 and day 21. For PD-L1 blockade, rat anti-mouse PD-L1 antibody was administered i.p. 5 times every 3 days, beginning on the day 14. Four differently treated groups were included in this experiment (Figure S5A). No increase of WHcAg-specific CD8 T cell response in the liver was observed in untreated, only ETV treated, and ETV plus PD-L1 blockade treated mice. In contrast, mice received ETV treatment in combination with DNA vaccination and PD-L1 blockade showed significantly increased WHcAg-specific CD8 T cell response in the liver (Figure S5B). We also monitored the change of WHV DNA levels in the serum of all mice. As expected, no significant change in the WHV DNA load throughout the experiment was observed in the untreated mice. Decrease of WHV DNA load was observed in all mice which received ETV treatment after day 7. On day 28, WHV DNA concentrations in only ETV treated mice returned to the pre-treatment levels. In contrast, significantly reduced WHV DNA levels were measured in mice received ETV plus PD-L1 blockade treatment and mice received triple combination therapy (Figure S5C). These results indicate that the triple combination regimen is able to break the immune tolerance against WHV antigens in WHV tg mice and leads to the suppression of viral replication.

## Discussion

The aim of therapeutic vaccination in chronic HBV infection is to elicit cellular immune responses specifically against HBV and thus reduce the viral burden. However, strategies investigated so far were often hampered by weak T cell responses observed after immunization, suggesting a strong need for alternative strategies to enhance T cell functions during chronic HBV infection. In this study, we evaluated the efficacy of antiviral treatment in combination with DNA vaccination and blockade of the inhibitory PD-1/PD-L1 pathway, which is a key factor of inducing T cell exhaustion during chronic viral infections in woodchucks chronically infected with WHV. Compared to nucleoside analogue treatment in combination with only therapeutic vaccine, additional PD-L1 blockade enhanced expansion and improved the function of WHcAg-specific CD8 T cells in woodchucks persistently infected with WHV. This triple combination therapy resulted in a prolonged suppression of WHV replication as compared to nucleoside analogue treatment alone or in combination with therapeutic vaccine. These results indicate that this may be a novel strategy for effective therapeutic vaccination of chronic HBV infection with subsequent complete recovery.

The quantity of antigen to which the immune system is exposed can induce different degrees of functional impairment of antiviral T cells, up to physical T cell deletion [15], [31]. This mechanism may play an important role in the HBV-specific T-cell hyporesponsiveness of chronic HBV infection, because high concentrations of antigens are constantly present at all stages of the infection. Studies have already confirmed that the functions of HBV-specific T cells are more profoundly inhibited in the presence of high viremia [16], [32], [33]. Therefore, when viral load is high and T cell dysfunction is severe, therapeutic vaccination alone can not induce a strong HBV-specific T cell response. Actually, poor responses and limited antiviral effects have been reported for the therapeutic vaccination of chronic HBV patients [7], [34], [35], [36]. In contrast, T cell exhaustion may be less severe at lower viral loads, and subsequent therapeutic vaccination may be more effective [9]. Studies of SIV infection support this concept because therapeutic vaccination is more effective after antiviral therapy [37], [38]. Therefore, the potent antiviral drug ETV was used in this study to strongly decrease both WHV DNA and WHsAg, which facilitated the induction of WHV-specific immune responses by DNA vaccination.

Another benefit of effective antiviral treatment is that it decreases PD-1 expression on CD8 T cells, which makes the rescue of exhausted T cell by PD-1/PD-L1 blockade possible. It has been recently clarified that the proportion of CD8 T cells expressing PD-1 and the levels of PD-1 on virus-specific T cells are strongly correlated with viral load in the plasma [17], [19], [20]. Antiretroviral treatment resulted in the dramatic decline of plasma viral load, coincident with a decrease in the PD-1 expression level on virus-specific CD8 T cells [17], [19]. In line with this, a better restoration of T cell functions upon *in vitro* anti-PD-L1 treatment was observed in chronic HBV patients with lower viremia [39]. In this study, we could not determine the PD-1 expression in WHV-specific T cells due to the lack of available woodchuck tetramers. Nevertheless, a significant positive correlation between the viral load and the PD-1 expression on total CD8 T cells in chronic WHV infection was observed. Both the proportion of PD-1+ CD8 T cells and the levels of PD-1 expression on CD8 T cells were significantly higher in the woodchucks with chronic WHV infection compared to naïve animals and resolvers. More importantly, during ETV treatment of those chronic carriers, a reduction of serum viral load was correlated with a dramatic decrease in the level of PD-1 expression on CD8 T cells. The PD-1^hi^ population of CD8 T cells observed in the chronic carriers completely disappeared after ETV treatment. It was reported that PD-1^hi^ CD8 T cells were not able to restore their function by PD-1/PD-L1 blockade in LCMV infection [40]. Therefore, the decrease of PD-1 expression on CD8 T cells caused by antiviral treatment may have increased the efficacy of *in vivo* PD-1/PD-L1 blockade on restoring CD8 T cell function. In contrast, treatment with PD-L1 antibody alone showed no effect on either enhancing T cell function or controlling WHV viral replication in chronic carriers (Figure S6). Due to the high costs of woodchucks and long experimental periods, we could not enroll enough woodchucks to establish exhaustive control groups (such as empty plasmid vaccine, et al) for this study. However, we did originally enroll one woodchuck as a control for examining irrelevant rabbit polyclonal antibody. This woodchuck received entecavir treatment, DNA vaccination and rabbit polyclonal IgG (Europa Bioproducts Ltd, Cambridge, England) injection. Compared to the woodchucks which received PD-L1 antibodies treatment, this woodchuck showed no significant increase in WHcAg-specific CD8 T cell responses in week one and two after antibody injection (Figure S7). Unfortunately, this woodchuck developed liver tumors during the experiment and died 4 weeks after antibody injection (week 28), which made us unable to tell the long term outcome of this animal.

One major concern of this triple regimen is whether antiviral treatment in combination with PD-L1 blockade alone is already enough for inducing virus-specific CD8 T cell response and controlling viral replication. To address this question, we examined the impact of the ETV treatment in combination with PD-L1 blockade alone on WHcAg-specific CD8 T cell response and the WHV replication in WHV tg mice. In comparison to the triple treatment, the dual treatment (ETV plus PD-L1 blockade) failed to induce detectable WHcAg-specific CD8 T cell response in the liver. Nevertheless, the dual treatment exhibited better effect on suppressing WHV replication than ETV treatment alone (Figure S5). This result indicates that PD-L1 blockade alone can give additive effect to ETV treatment on suppressing WHV replication, but DNA vaccination seems to be essential for achieving a robust and persistent viral-specific CD8 T cell response in chronic hepatitis infection.

DNA vaccination is an attractive method for the treatment of chronic infection and tumors. Clinical studies provided proof of concept that DNA vaccines could safely induce immune responses (albeit low-level responses) in humans. In this study, in total 12 DNA vaccinations were performed to elicit WHV-specific CD8+ T-cell responses to in WHV chronic carriers. Many improvements have been incorporated into the new DNA vaccine strategies, including optimization of antigen expression on a per cell basis and improved formulation to enhance and direct immune responses [41]. We have also showed very recently that a DNA prime-adenovirus (AdV) boost vaccination regimen could elicit strong and specific CD8+ T-cell responses to WHcAg in mice and woodchucks [30]. This strategy induces more robust T cell responses than the conventional DNA vaccine does with even less injections. Unfortunately, this new regimen was not available when we performed the presented PD-L1 blockade experiment. Therefore, enrolling more efficient DNA vaccine protocols such as the DNA prime-AdV boost protocol to our current combination therapy strategy may be an interesting option in future trials.

In this study and our previous study [30], we immunized ETV treated woodchucks with two DNA plasmids which express WHcAg and WHsAg respectively. In contrast to the WHcAg-specific T cell response, the WHsAg-specific CD8 T cell response induced by DNA vaccination in ETV treated woodchucks was weak. This could be due to 2 possible reasons: (1) The WHcAg expressing plasmid pCGWHc is more efficient at antigen expression than the WHsAg expressing plasmid pWHsIm [29]. Improved WHcAg expression may lead to the induction of a more T cell response *in vivo*. (2) The ongoing secretion of high amounts of WHsAg in the circulation of woodchucks may have hampered the induction of a vigorous WHsAg-specific CD8 T cell response. Nevertheless, two woodchucks of the PD-L1 blockade group developed anti-WHs antibodies, indicating that WHsAg vaccine may also contribute to the control of WHV infection. In addition, a vigorous T-cell response against HBcAg is crucial for the resolution of the infection but is predominantly absent in chronic hepadnaviral infections. Thus, using T cell vaccines targeting the core protein seems to be essential for a potent therapeutic strategy. The observation in this study suggests the conclusion that the T cell tolerance against WHcAg is easier to be broken than the tolerance against WHsAg in chronic carriers by DNA vaccines, and the induced robust WHcAg-specific CD8 T cell response contributes greatly to the control of WHV infection.

A major concern of using PD-1/PD-L1 pathway blockade treatment of chronic HBV infection is that this treatment may lead to severe liver inflammation and may result in fulminant hepatitis. However, we did not observe any increase of liver inflammation in the animals after the PD-L1 antibody treatment by monitoring GOT levels. In most of the treated woodchucks, the GOT flares appeared at the stage of ETV treatment alone or the beginning of DNA vaccination. Consistent with our results, recent studies of clinic phase 1 trials have shown the safety of using anti-PD-L1 and anti-PD1 antibodies in cancer patients treatment [42], [43].

The negative regulation of T-cell function involves numerous receptor and ligand interactions in separate cellular compartments at different phases of the immune response. Recent studies have suggested that multiple inhibitory receptors, such as CTLA-4 [44], TIM-3 [45] and LAG-3 [46], have played important roles in T-cell exhaustion during persistent HBV infection. These observations suggest that there may be a synergy between blockade of the various inhibitory pathways and encourage the examination of combinatorial strategies for treatment of woodchucks with chronic WHV or to later patients with chronic HBV in the future. Moreover, it has been shown very recently that activating Toll-like receptor 7 signaling can induce clearance of HBV-infected cells and prolonged suppression of HBV replication in chronically infected chimpanzees [47]. The mechanism of activating immune system and suppressing HBV by using TLR agonist significantly differs from that of PD-1/PD-L1 pathway blockade in T cells. Therefore, combining TLR7 agonist and PD-L1 antibody treatment may activate different arms of immune system and may achieve synergistic effects on control of chronic HBV infection.

WHV and HBV show a marked similarity in the virion structure, genomic organization, mechanism of replication, and host immune responses during infection and recovery. Woodchucks chronically infected with WHV develop progressively severe hepatitis, which is remarkably similar to what is observed in chronic HBV patients. Likewise, the results of antiviral drug efficacy and toxicity studies in the woodchucks chronically infected with WHV are predictive for responses of chronic HBV patients. However, we are aware that we can not predict whether the triple regimen of treatment described in this paper will be successful in patients with chronic HBV.

In conclusion, our study for the first time demonstrates that antiviral treatment in combination with therapeutic vaccination and PD-L1 blockade potently boosts virus-specific CD8 T cell responses and promotes viral control during chronic hepadnavirus infection. Further studies in a larger number of chronic carriers need to be conducted for investigating whether the rate of virus elimination could be significantly enhanced. Our results may lay a foundation for initiating a dual therapy (combination of nucleotide analogue and PD-L1 blockade) or a triple therapy (combination of nucleotide analogue, DNA vaccine and PD-L1 blockade) in chronic HBV patients.

## Materials and Methods

### Ethical statement

All animal experiments were conducted in accordance with *the Guide for the Care and Use of Laboratory Animals* and were approved by the local Animal Care and Use Committee (Animal Care Center, University of Duisburg-Essen, Essen, Germany and the district government of Düsseldorf, Germany; permission numbers G1303/12 and G1304/12). The experiments were performed under ketamine-xylazine anesthesia, and all efforts were made to minimize suffering.

### Woodchucks and mice

The woodchucks (Marmota monax) were purchased from North Eastern Wildlife (Harrison, ID). WHV chronic carriers were captive-born 1-year-old woodchucks neonatally infected with WHV. Persistence of WHV infection was based on the consecutive detection of WHV DNA and WHsAg in serum starting at 3 months of age. WHV transgenic mice lineage carrying WHV wild-type (strain 1217) was created on C57BL/6 background (genotype H-2b/b) and previously characterized [30]. Animals were maintained according to the guidelines of the animal facility at the University Hospital Essen.

### Cloning of woodchuck PD-1

Total RNA was extracted from fresh isolated woodchuck PBMCs using the TRIZOL reagent (Invitrogen) according to the manufacturer's instructions and subjected to RT-PCR for amplification of cDNAs of woodchuck PD-1. The primers used for cloning woodchuck PD-1 cDNAs were: 5′-ATG CAG GGC CGG TGG-3′, 5′-GCC TGG AAG CTG GCC T-3′. The specific PCR fragments were cloned into pMD18-T TA vector (TAKARA) and subjected to DNA sequencing. The secondary structure of protein sequences data was predicted by online service of the Swiss Institute of Bioinformatics (http://swissmodel.expasy.org).

### Quantitative real-time RT-PCR

Sequences of PCR primer pairs for woodchuck PD-1 and β-actin are as follows: PD-1 (forward, 5′-AGC CCC AGC AAG CAG AAC-3′; reverse, 5′-GCC CCG CAG AGG TAG AGG-3′) and β-actin (forward: 5′-TGG AAT CCT GTG GCA TCC ATG AAA C-3′; reverse, 5′-TAA AAC GCA GCT CAG TAA CAG TCC G-3′). The quantification of woodchuck PD-1 mRNAs was performed by real time RT-PCR using QuantiFast SYBR Green RT-PCR Kit (Invitrogen, Karlsruhe, Germany) on a Light Cycler. WHV DNA was quantified by real-time PCR as described previously [28].

### Flow cytometry

Cell-surface and intracellular staining for flow cytometry analysis was performed using BD Biosciences or eBioscience reagents as described previously [28], [48]. For woodchuck PD-1 and CD3 staining, an anti-mouse PD-1 FITC-conjugated antibody (clone J43, eBioscience) and an anti-rat CD3 PE-conjugated antibody (clones G4.18, BD Biosciences) were used. Data were acquired using a FACSCalibur flow cytometer (BD Biosciences, Heidelberg, Germany) and analyzed using FlowJo software (Tree Star, Inc., Ashland, Oregon). Cell debris and dead cells were excluded from the analysis based on scatter signals and 7-Amino-actinomycin D fluorescence.

### ETV treatment of chronically WHV-infected woodchucks

Chronically WHV-infected woodchucks were treated for 28 weeks with the nucleoside analogue entecavir (ETV, Bristol-Myers Squibb, New York, NY). Initially, the drug was administered for 12 weeks by using osmotic pumps (DURECT, Cupertino, CA) which were implanted surgically under the skin of the animals. The pump releases subcutaneously approximately 0.2 mg of ETV per day. Pumps were exchanged every 4 weeks and overall 3 pumps were implanted for each woodchuck. From week 10 to 28, subcutaneous injections of 1.5 mg of ETV were performed weekly.

### DNA vaccination

The protocol of the immunization of woodchucks was described previously [49]. The immunizations with previously constructed DNA plasmids expressing WHcAg and WHsAg [29] were performed by intramuscular injection. A week prior to the injection of plasmids, 500 µl of cardiotoxin (Latoxan, Valence, France) at a concentration of 10 µM in PBS was injected into *M. tibialis cranialis* of woodchucks. Woodchucks were vaccinated 12 times by intramuscular injection of 500 µl of plasmids into each *M. tibialis cranialis* at the indicated time points. For WHV transgenic mice immunization, ten to twelve weeks old sex-matched groups of mice were pretreated by intramuscular injection of 50 µl of cardiotoxin into Tibialis anterior muscle one week before the plasmid immunization. Animals were then intramuscularly vaccinated twice with 100 µg of pCGWHc (50 µg per muscle) at two weeks interval.

### *In vivo* PD-L1 blockade

Polyclonal rabbit anti-woodchuck PD-L1 antibody was generated by our lab as described previously [24]. At week 24 of the ETV therapy, woodchuck PD-L1 antibody (25 mg/kg) in PBS was intravenously injected to 3 woodchucks. Administration of rabbit isotype antibody to 1 woodchuck served as a control. Antibodies were injected every 2 days, and were overall injected 3 times. For mice PD-L1 blockade, 200 µg of rat anti-mouse PD-L1 antibody (10F:9G2) was administered i.p. 5 times every 3 days.

### CD107a degranulation assay of woodchuck PBMCs

CD107a degranulation assay was performed as described previously [28]. Briefly, woodchuck PBMCs were separated by Ficoll density gradient centrifugation and stimulated with 2 µg/ml WHcAg-derived epitope c96-110 (KVRQSLWFHLSCLTF). Unstimulated cells and cells stimulated with 2 µg/ml of a control CMV-derived peptide (YILEETSVM) served as negative controls. After 3 days of cultivation, cells were restimulated with corresponding peptides and stained for CD107a for 5 hours. Data were acquired using a FACSCalibur flow cytometer.

### Proliferation of woodchuck PBMCs

Antigen-specific proliferation of woodchuck PBMCs was determined by 2[^3^H]-adenine-based assay as described previously [49]. Briefly, 5×10^4^ PBMCs were stimulated with 5 µg/ml purified WHcAg protein for 5 days. Unstimulated cells served as a negative control. Afterwards, cells were labelled with 1 µCi of 2[^3^H]-adenine (Hartmann Analytic, Braunschweig, Germany) for 16 h and collected using a cell harvester (Perkin Elmer, Waltham, MA). Results for triplicate cultures are presented as a mean stimulation index (SI). SI is calculated with the formula: (stimulated cpm - blank cpm)/(unstimulated cpm - blank cpm).

### Serology

Woodchuck anti-WHs antibodies were detected by enzyme-linked immunosorbent assay (ELISA) as described previously [49], [50].

### Quantification of WHV DNA in the serum

WHV DNA was quantified by real-time PCR using Platinum SYBR Green Kit (Invitrogen) as described previously [28].

### Analysis of WHV replication intermediates in liver tissues

Total DNA from liver samples of chronically WHV-infected woodchucks was extracted using the QIAamp Tissue Kit (Qiagen, Hilden, Germany) according to the manufacturer's instructions. WHV replication intermediates were analyzed by Southern blot hybridization as described previously [51], [52]. WHV DNA was determined by both qualitative PCR and quantitative real-time PCR using Platinum SYBR Green Kit (Invitrogen). WHV cccDNA was determined by PCR as described previously [53].

### Determination of WHsAg concentration in woodchuck sera

Serum WHsAg concentration was determined by electroimmunodiffusion as described previously [23].

### Evaluation of GOT levels

The glutamic oxaloacetic transaminase (GOT; also known as aspartate transaminase, AST) level was quantified according to the standard diagnostic procedure at the Central Laboratory of University Hospital Essen. The values above 50 IU (international units) per millilitre were considered as elevated.

### Statistical analysis

Statistics comparing two groups were done using the nonparametric *t* test. When more than two groups were compared, a one-way ANOVA was used with a Tukey posttest (GraphPad Prism software; GraphPad, San Diego, CA).

## Supporting Information

Figure S1 **Effect of** ***in vivo*** **PD-L1 blockade on enhancing WHsAg-specific CD8 T cell immunity.** WHsAg-specific T cell responses of differently treated woodchucks were analyzed by CD107a degranulation assay. The kinetics of WHsAg-specific CD8 T cell response of 4 differently treated groups of woodchucks (n = 3) is presented. **C**: control group without any treatment; **E**: ETV treated only group; **ED**: ETV in combination with DNA vaccinations; **EDA**: ETV and DNA vaccination in combination with anti-PDL1 antibody treatment.
(TIFF)

Figure S2 **Development of massive HCC in woodchuck EDA3.** Autopsy was performed after the sacrifice of the woodchuck. Red arrows indicate the HCC nodules in the liver of the woodchuck.
(TIFF)

Figure S3 **Effect of** ***in vivo*** **PD-L1 blockade on enhancing control of WHV replication.** The kinetics of serum WHsAg concentrations of woodchucks with triple combination treatment is presented individually.
(TIF)

Figure S4 **Determination of sera WHcAb levels in triple combination treated woodchucks.** The kinetics of serum anti-WHc antibodies levels of woodchucks with triple combination treatment is presented. Anti-WHc antibodies level was determined using the following formula: S/N ratio = sample OD value/negative control OD value. Cut-off value of S/N ratio was 2.1.
(TIF)

Figure S5 ***In vivo*** **PD-L1 blockade in combination with therapeutic vaccination to enhance WHV-specific T cell immunity and to control WHV replication in WHV tg mice.** (A) Schema of triple combination therapy of ETV treatment, DNA vaccination and PD-L1 blockade in WHV tg mice. The antiviral drug ETV was daily administered for 10 days to suppress the WHV replication in mice. For PD-L1 blockade, 200 µg of rat anti-mouse PD-L1 antibody (10F:9G2) was administered i.p. 5 times every 3 days, beginning on the day 14. For DNA vaccination, mice received 2 times intramuscular immunizations with DNA plasmid expressing WHcAg on day 7 and day 21. Four groups of mice were included. **C**: control group without any treatment; **E**: ETV treated only group; **EA**: ETV in combination with anti-PDL1 antibody treatment group; **EDA**: ETV and DNA vaccination in combination with anti-PDL1 antibody treatment group. (B) WHcAg-specific CD8 T cell responses in the liver of differently treated mice were analyzed by intracellular cytokine staining. (C) Serum WHV DNA concentrations of different treatment groups are presented at indicated time points.
(TIF)

Figure S6 **Determination of the WHV-specific T cell responses and WHV viral loads in woodchucks with only** ***in vivo*** **PD-L1 blockade.** Woodchuck PD-L1 antibody (25 mg/kg) in PBS was intravenously injected to 3 woodchucks with chronic WHV infection. Antibodies were injected every 2 days, and were overall injected 3 times. (A) WHcAg-specific T cell responses of treated woodchucks were analyzed by CD107a degranulation assay (left) and proliferation assay (right). (B) Serum WHV DNA concentrations of treated woodchucks are presented at indicated time points.
(TIF)

Figure S7 **Determination of the WHV-specific T cell responses in woodchuck received rabbit isotype antibody treatment.** One woodchuck received ETV treatment, DNA vaccinations and rabbit isotype antibody injection. WHcAg-specific T cell responses of treated woodchuck were analyzed by CD107a degranulation assay (left) and proliferation assay (right).
(TIF)

Table S1 **Raw data of proliferation assay of woodchucks with triple combination treatment.** Antigen-specific proliferation of woodchuck PBMCs was determined by 2[^3^H]-adenine-based assay as described previously. 5×10^4^ PBMCs were stimulated with 5 µg/ml purified WHcAg protein for 5 days. Unstimulated cells served as a negative control. The CPM values of the assay are presented at indicated time points.
(DOC)
