# Supplementary figures and images for: Enhancing Virus-Specific Immunity In Vivo by Combining Therapeutic Vaccination and PD-L1 Blockade in Chronic Hepadnaviral Infection
Source: PLoS Pathog. 2014 Jan 2;10(1):e1003856. doi: 10.1371/journal.ppat.1003856 (PMC3879364; doi:10.1371/journal.ppat.1003856)

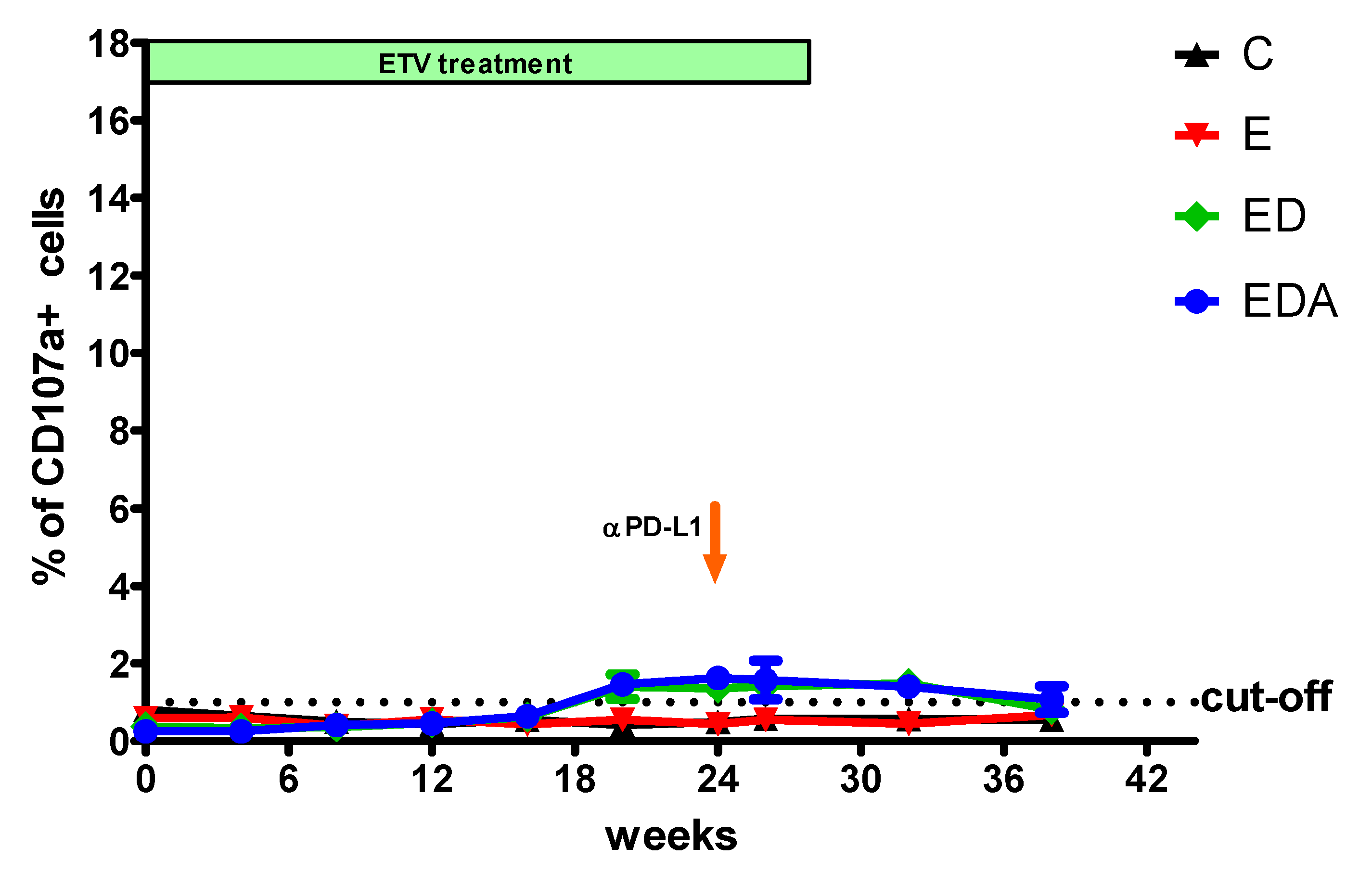

Supplement: Figure S1 — Effect of in vivo PD-L1 blockade on enhancing WHsAg-specific CD8 T cell immunity. WHsAg-specific T cell responses of differently treated woodchucks were analyzed by CD107a degranulation assay. The kinetics of WHsAg-specific CD8 T cell response of 4 differently treated groups of woodchucks (n = 3) is presented. C: control group without any treatment; E: ETV treated only group; ED: ETV in combination with DNA vaccinations; EDA: ETV and DNA vaccination in combination with anti-PDL1 antibody treatment. (TIFF) [file ppat.1003856.s001.tiff]

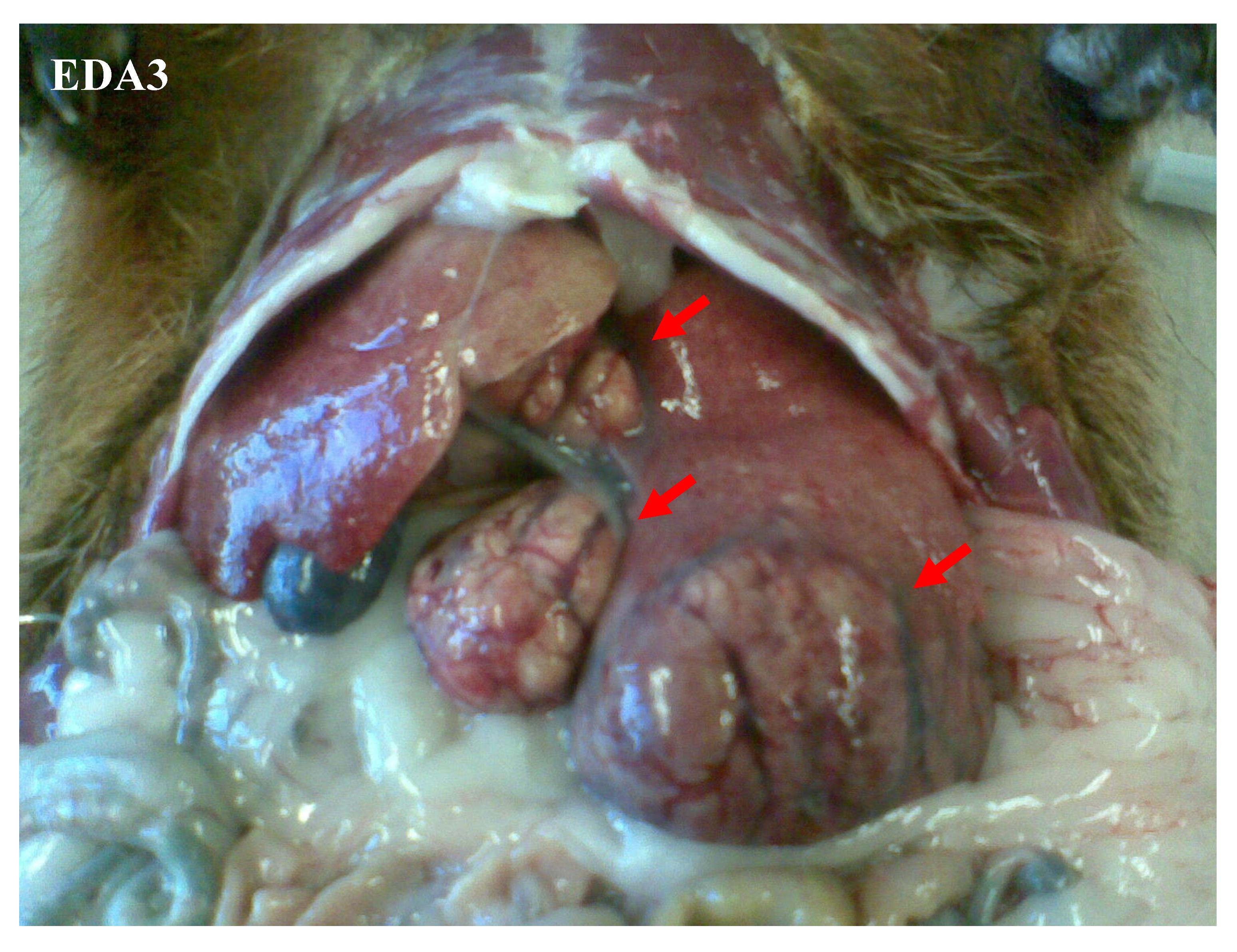

Supplement: Figure S2 — Development of massive HCC in woodchuck EDA3. Autopsy was performed after the sacrifice of the woodchuck. Red arrows indicate the HCC nodules in the liver of the woodchuck. (TIFF) [file ppat.1003856.s002.tiff]

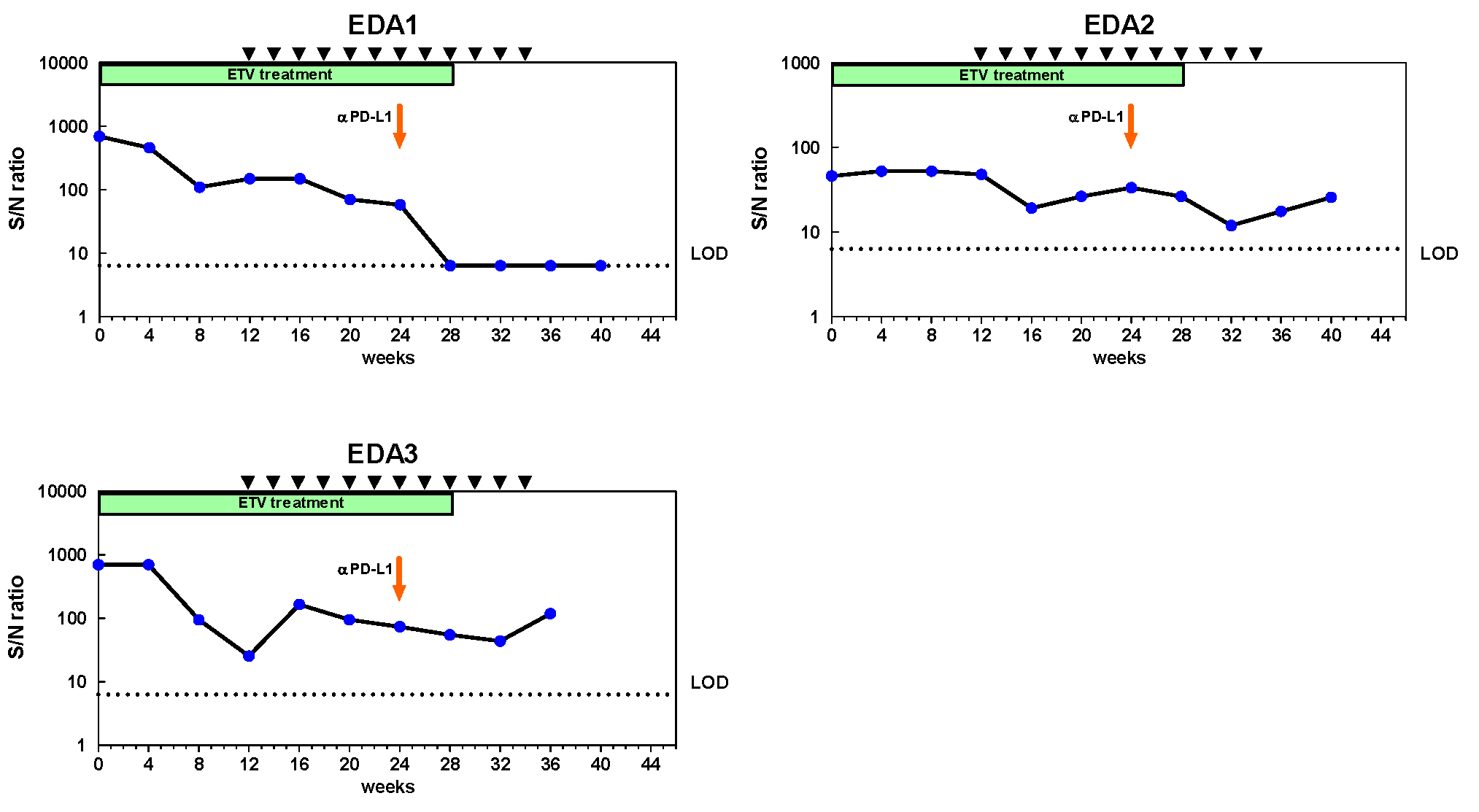

Supplement: Figure S3 — Effect of in vivo PD-L1 blockade on enhancing control of WHV replication. The kinetics of serum WHsAg concentrations of woodchucks with triple combination treatment is presented individually. (TIF) [file ppat.1003856.s003.tif]

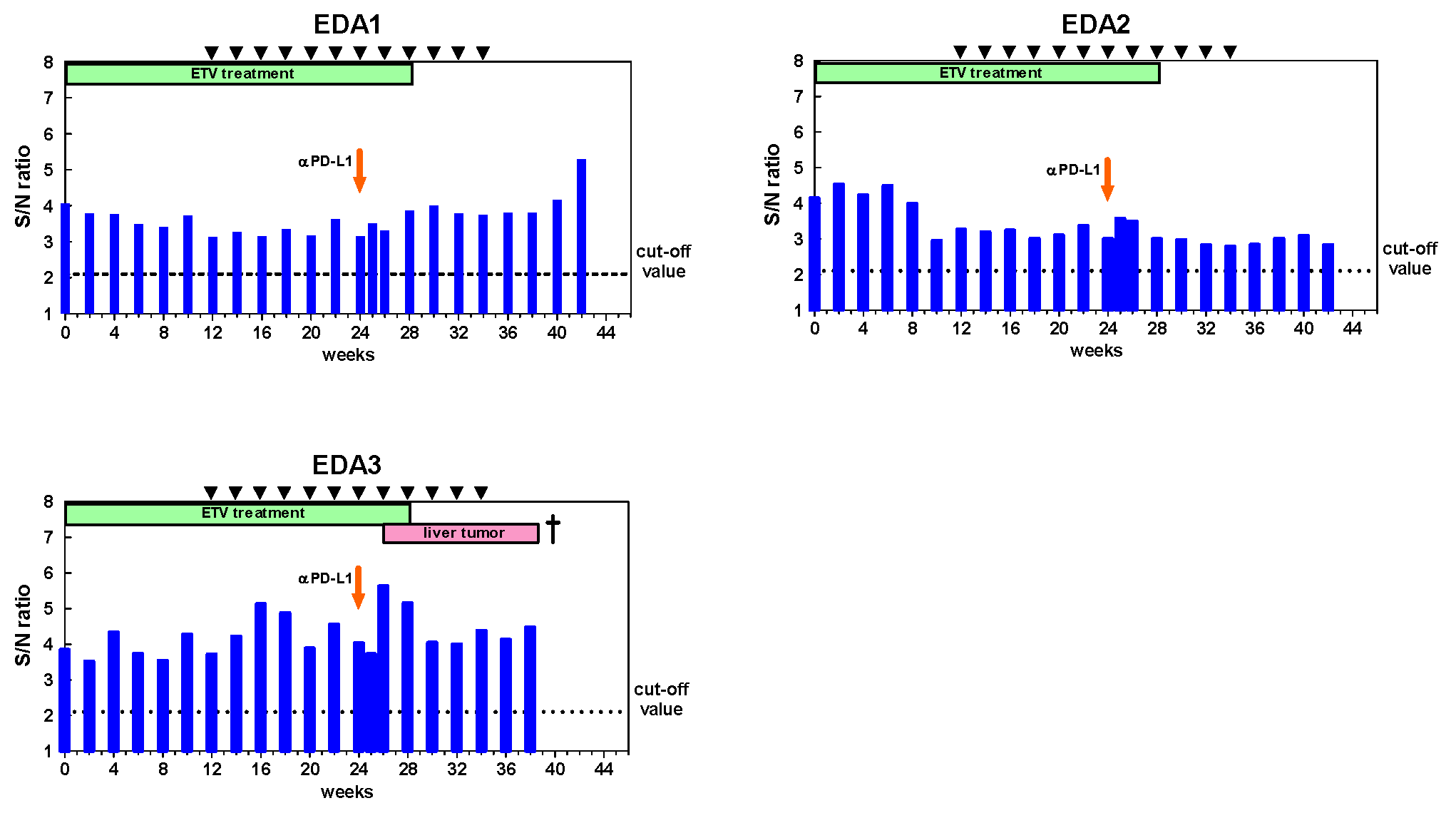

Supplement: Figure S4 — Determination of sera WHcAb levels in triple combination treated woodchucks. The kinetics of serum anti-WHc antibodies levels of woodchucks with triple combination treatment is presented. Anti-WHc antibodies level was determined using the following formula: S/N ratio = sample OD value/negative control OD value. Cut-off value of S/N ratio was 2.1. (TIF) [file ppat.1003856.s004.tif]

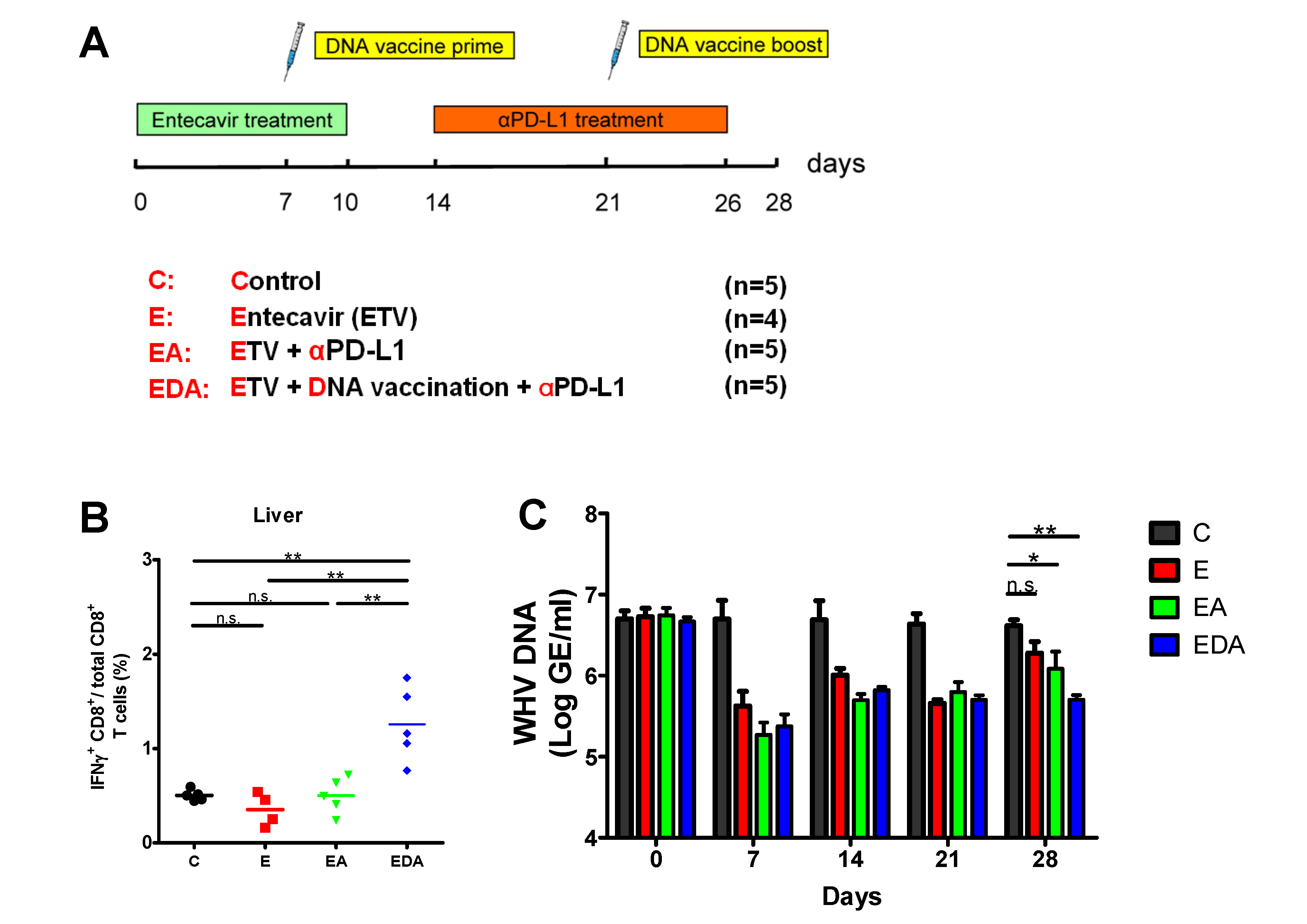

Supplement: Figure S5 — In vivo PD-L1 blockade in combination with therapeutic vaccination to enhance WHV-specific T cell immunity and to control WHV replication in WHV tg mice. (A) Schema of triple combination therapy of ETV treatment, DNA vaccination and PD-L1 blockade in WHV tg mice. The antiviral drug ETV was daily administered for 10 days to suppress the WHV replication in mice. For PD-L1 blockade, 200 µg of rat anti-mouse PD-L1 antibody (10F:9G2) was administered i.p. 5 times every 3 days, beginning on the day 14. For DNA vaccination, mice received 2 times intramuscular immunizations with DNA plasmid expressing WHcAg on day 7 and day 21. Four groups of mice were included. C: control group without any treatment; E: ETV treated only group; EA: ETV in combination with anti-PDL1 antibody treatment group; EDA: ETV and DNA vaccination in combination with anti-PDL1 antibody treatment group. (B) WHcAg-specific CD8 T cell responses in the liver of differently treated mice were analyzed by intracellular cytokine staining. (C) Serum WHV DNA concentrations of different treatment groups are presented at indicated time points. (TIF) [file ppat.1003856.s005.tif]

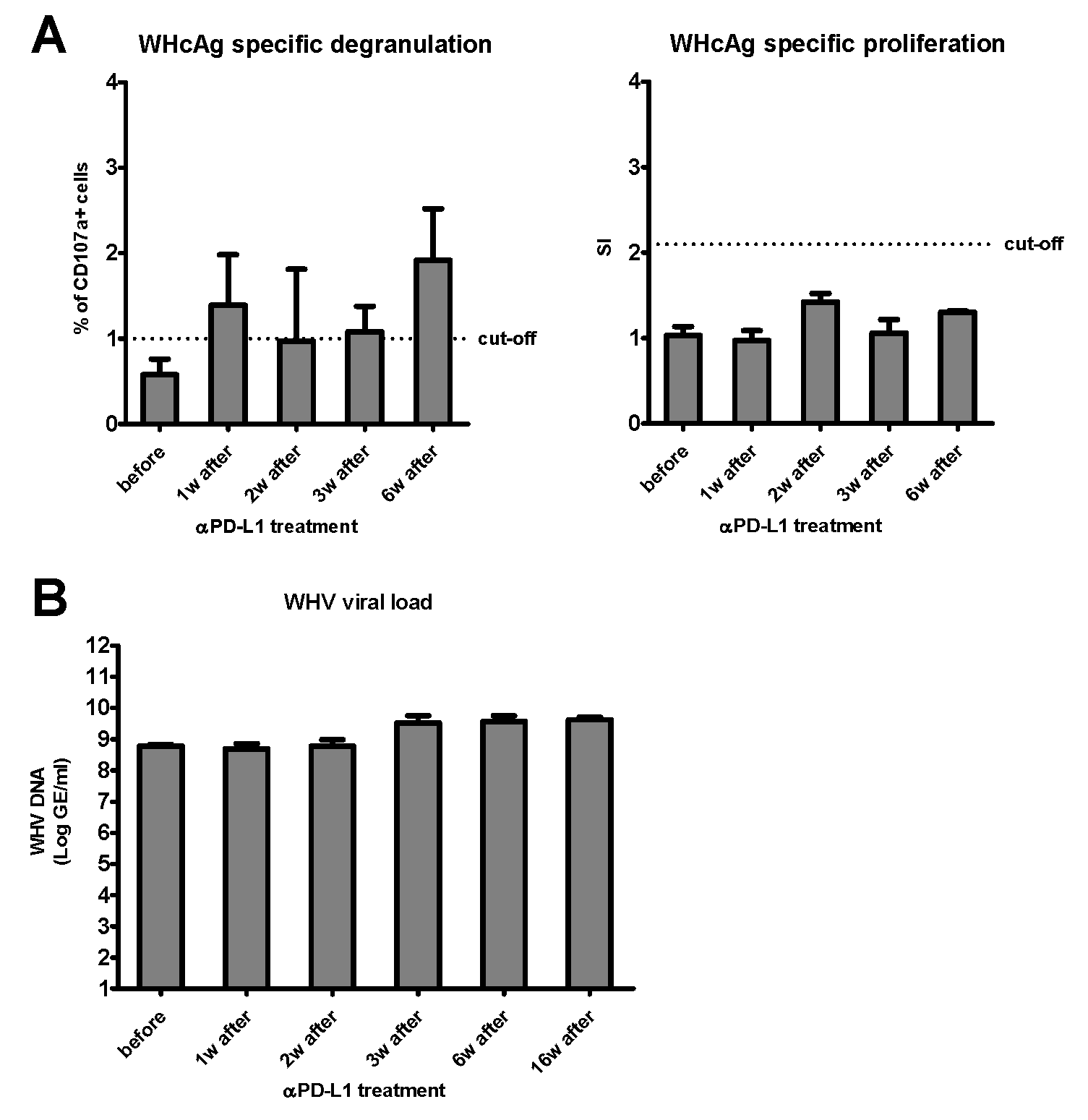

Supplement: Figure S6 — Determination of the WHV-specific T cell responses and WHV viral loads in woodchucks with only in vivo PD-L1 blockade. Woodchuck PD-L1 antibody (25 mg/kg) in PBS was intravenously injected to 3 woodchucks with chronic WHV infection. Antibodies were injected every 2 days, and were overall injected 3 times. (A) WHcAg-specific T cell responses of treated woodchucks were analyzed by CD107a degranulation assay (left) and proliferation assay (right). (B) Serum WHV DNA concentrations of treated woodchucks are presented at indicated time points. (TIF) [file ppat.1003856.s006.tif]

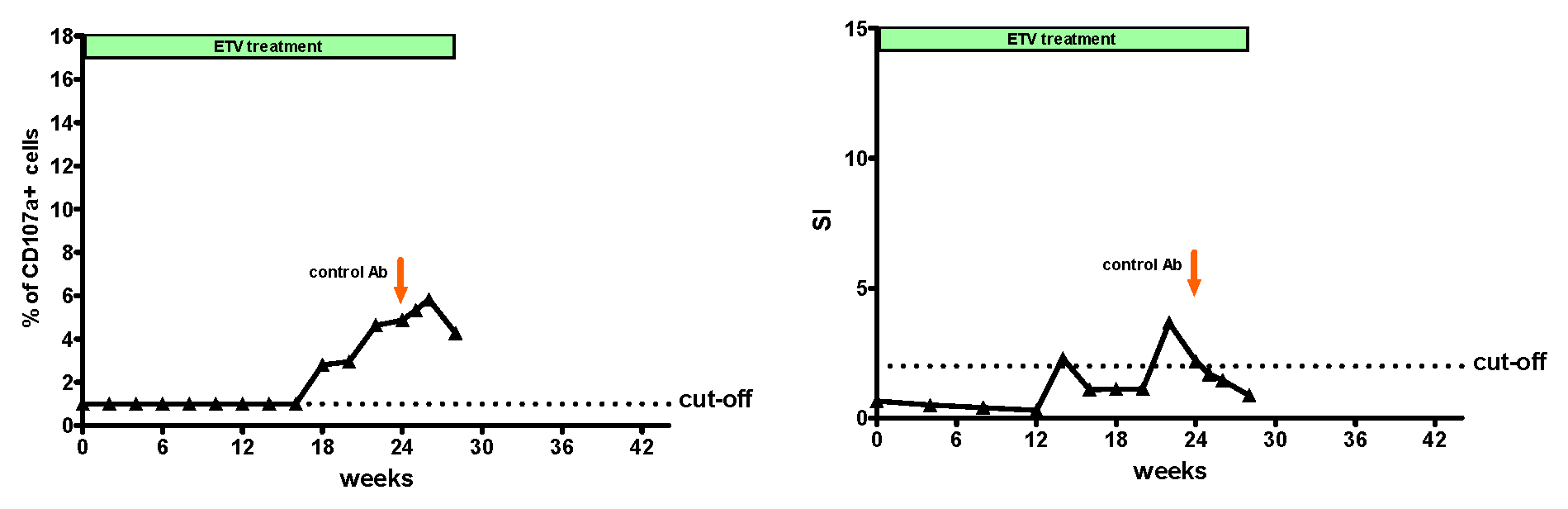

Supplement: Figure S7 — Determination of the WHV-specific T cell responses in woodchuck received rabbit isotype antibody treatment. One woodchuck received ETV treatment, DNA vaccinations and rabbit isotype antibody injection. WHcAg-specific T cell responses of treated woodchuck were analyzed by CD107a degranulation assay (left) and proliferation assay (right). (TIF) [file ppat.1003856.s007.tif]
