# Supplementary material for: Enhancing Virus-Specific Immunity In Vivo by Combining Therapeutic Vaccination and PD-L1 Blockade in Chronic Hepadnaviral Infection
Source: PLoS Pathog. 2014 Jan 2;10(1):e1003856. doi: 10.1371/journal.ppat.1003856 (PMC3879364; doi:10.1371/journal.ppat.1003856)
Supplement: Table S1 — Raw data of proliferation assay of woodchucks with triple combination treatment. Antigen-specific proliferation of woodchuck PBMCs was determined by 2[3H]-adenine-based assay as described previously. 5×10^4^ PBMCs were stimulated with 5 µg/ml purified WHcAg protein for 5 days. Unstimulated cells served as a negative control. The CPM values of the assay are presented at indicated time points. (DOC) [file ppat.1003856.s008.doc]

**Supplementary Material**

**Table S1. Raw data of proliferation assay of woodchucks with triple combination treatment.**

| Weeks | EDA1 | | | EDA2 | | | EDA3 | | |
| --- | --- | --- | --- | --- | --- | --- | --- | --- | --- |
|  | control | WHcAg | SI | control | WHcAg | SI | control | WHcAg | SI |
| 0 | 1090 | 967 | 0.91 | 1433 | 865 | 0.88 | 968 | 767 | 0.78 |
|  | 907 | 714 |  | 1208 | 1003 |  | 1017 | 812 |  |
|  | 765 | 820 |  | 941 | 1298 |  | 1042 | 786 |  |
| 4 | 1208 | 688 | 0.47 | 1402 | 1098 | 0.70 | 1222 | 1001 | 0.83 |
|  | 1540 | 539 |  | 1133 | 879 |  | 1433 | 995 |  |
|  | 1322 | 701 |  | 1608 | 903 |  | 1109 | 1122 |  |
| 8 | 1418 | 862 | 0.56 | 1608 | 877 | 0.58 | 1590 | 1044 | 0.74 |
|  | 1355 | 794 |  | 1227 | 679 |  | 1432 | 1248 |  |
|  | 1603 | 778 |  | 1508 | 943 |  | 1398 | 989 |  |
| 12 | 1716 | 3784 | 2.58 | 1490 | 1807 | 1.53 | 1042 | 989 | 0.88 |
|  | 1865 | 5232 |  | 1187 | 1809 |  | 1211 | 1055 |  |
|  | 1788 | 4848 |  | 1203 | 2311 |  | 1114 | 912 |  |
| 16 | 1120 | 3027 | 2.47 | 802 | 1647 | 1.75 | 1866 | 1465 | 0.78 |
|  | 1056 | 2080 |  | 912 | 1263 |  | 1650 | 1208 |  |
|  | 1098 | 2976 |  | 1020 | 1886 |  | 1698 | 1412 |  |
| 20 | 620 | 2464 | 3.05 | 1044 | 3377 | 3.17 | 2498 | 4772 | 2.29 |
|  | 665 | 1512 |  | 1280 | 3865 |  | 1808 | 6043 |  |
|  | 708 | 2108 |  | 1330 | 4328 |  | 1976 | 3544 |  |
| 24 | 1194 | 1717 | 1.36 | 4353 | 9117 | 2.78 | 2146 | 6635 | 2.39 |
|  | 1064 | 1363 |  | 2631 | 10217 |  | 1847 | 2919 |  |
|  | 1129 | 1540 |  | 3492 | 9767 |  | 1997 | 4777 |  |
| 26 | 3193 | 9090 | 4.44 | 1292 | 2431 | 2.37 | 1665 | 9526 | 3.97 |
|  | 2566 | 16483 |  | 1538 | 4275 |  | 2240 | 8296 |  |
|  | 2880 | 12787 |  | 1416 | 3360 |  | 2814 | 8862 |  |
| 30 | 1573 | 5671 | 3.44 | 270 | 673 | 2.03 | 941 | 807 | 0.99 |
|  | 1662 | 5467 |  | 614 | 1118 |  | 618 | 738 |  |
|  | 1620 | 5570 |  | 442 | 898 |  | 780 | 773 |  |
| 34 | 820 | 3990 | 3.70 | 471 | 6863 | 12.94 | 562 | 613 | 0.74 |
|  | 1063 | 3000 |  | 461 | 5198 |  | 1765 | 1115 |  |
|  | 952 | 3496 |  | 466 | 6032 |  | 1164 | 864 |  |
| 38 | 803 | 5301 | 7.58 | 757 | 5041 | 18.38 | n.d. | n.d. | n.d. |
|  | 875 | 6412 |  | 327 | 14887 |  |  |  |  |
|  | 840 | 7365 |  | 542 | 9965 |  |  |  |  |

Antigen-specific proliferation of woodchuck PBMCs was determined by 2[^3^H]-adenine-based assay as described previously. 5×10^4^ PBMCs were stimulated with 5 µg/ml purified WHcAg protein for 5 days. Unstimulated cells served as a negative control. The CPM values of the assay are presented at indicated time points.
